# Measuring sleep disturbance in advanced cancer using the brief Pittsburgh sleep quality index (bPSQI)

**DOI:** 10.1093/sleepadvances/zpag018

**Published:** 2026-02-09

**Authors:** Craig Gouldthorpe, Andrew Davies

**Affiliations:** School of Medicine, Trinity College Dublin, Dublin, Ireland; Academic Department of Palliative Medicine, Our Lady’s Hospice & Care Services, Dublin, Ireland; School of Medicine, Trinity College Dublin, Dublin, Ireland; Academic Department of Palliative Medicine, Our Lady’s Hospice & Care Services, Dublin, Ireland

**Keywords:** sleep quality, cancer, insomnia, accelerometry

## Abstract

Sleep disturbance is common among patients with cancer and is linked to significant morbidity, poorer quality of life and reduced survival in this population. The Pittsburgh Sleep Quality Index (PSQI) can identify poor sleep quality in this population, although a higher threshold value may be required compared to the general population. The brief PSQI (bPSQI), consisting of six of the original 19 items, offers a quicker and simpler tool. The bPSQI has demonstrated comparable accuracy in identifying poor sleep in the general population but remains unexplored in patients with advanced cancer. This observational study of 65 patients with advanced cancer reiterates the prevalence of sleep disturbance, demonstrates good internal consistency of the bPSQI and notes higher bPSQI scores with increasing subjective sleep-related distress. Although significant associations were noted between the bPSQI global score, individual bPSQI item scores and single-item sleep disturbance questions, the study cautions against the use of single-item questions in detecting sleep problems. Discrepancies were noted between subjective and objective sleep assessments. The findings support a higher threshold value for identifying poor sleep quality using the bPSQI. Although limited by a small sample size, the findings emphasize the need for further validation of the bPSQI in this population and to ensure that assessment methods align with research aims.

## Introduction

Sleep disturbance, which may describe difficulty initiating or maintaining sleep, a change in sleep timing, or excessive daytime sleepiness, is common among patients with cancer (combined prevalence - 60.7%; 95% CI = 58.1% to 63.3%), and especially among patients with advanced cancer (combined prevalence - 70.8%; 95% CI = 61.7% to 78.5%) [1]. Furthermore, sleep disturbance is associated with significant morbidity, reduced quality of life, and reduced survival in this population [2, 3]. Sleep disturbance in patients with cancer is often multifactorial, with clear links to the underlying cancer and its pro-inflammatory state, various cancer treatments and cancer-related symptoms [3].

Sleep disturbance is included in several generic assessment tools (e.g. Memorial Symptom Assessment Scale–Short Form (MSAS-SF), and European Organization for Research and Treatment of Cancer Quality of Life Questionnaire (EORTC-QLQ-C30). Sleep specific assessment tools are rarely used in clinical practice, and in research involving patients with advanced cancer, the Pittsburgh Sleep Quality Index (PSQI) is often used [4]. The PSQI contains 19 self-rated items to assess sleep quality over the previous month, and a “total” score of >5 has been used to identify those with poor sleep quality in the general population [4]. However, in patients with cancer, a threshold score > 8 may be more appropriate at detecting sleep problems [5].

The brief PSQI (bPSQI, Table 1), comprised of six of the original PSQI questions, results in 5 scored items and a score of 0-15, whereby higher scores represent poorer sleep. Although the bPSQI demonstrates similar sensitivity (75.82%) and specificity (76.99%) in identifying poor sleepers in the general population (using a cut off value of >5), there is no published evidence supporting validity of the bPSQI in patients with advanced cancer [6]. The brevity and simpler scoring of the bPSQI, if proven a valid tool, would be of benefit in the clinical setting to reduce burden to clinicians and patients alike, and to aid in identifying a clinical prevalent and relevant condition in patients with advanced cancer. This study aimed to assess the bPSQI, against single-item sleep disturbance questions and objective sleep assessments, in a cohort of patients with advanced cancer.

**Table 1:** The brief Pittsburgh Sleep Quality Index (bPSQI)

During the past month				
What time have you usually gone to bed at night?				
What time have you usually gotten up in the morning?				
How long (in minutes) has it usually takes you to fall asleep each night?				
How many hours of actual sleep did you get at night?				
Have you had trouble sleeping because you wake up in the middle of the night or early morning?	(Not during the past month)	(Less than once a week)	(Once or twice a week)	(Three or more times a week)
How would you rate your sleep quality overall?	(Very good)	(Fairly good)	(Fairly bad)	(Very bad)
**Scored items**				
**Sleep quality:** Very good = 0; Fairly good = 1; Fairly bad = 2; Very bad = 3				
**Sleep disturbance:** Not during the past month = 0; Less than once a week = 1, Once or twice a week = 2, Three or more times a week = 3				
**Sleep onset latency** (minutes): ≤15 = 0; 16–30 = 1; 31–60 = 2; >60 = 3				
**Sleep duration** (hours): >7 = 0; 6–7 hours = 1; 5–6 = 2; <5 = 3				
**Sleep efficiency** (sleep duration / time in bed): ≥85% = 0, 75%–84% = 1, 65%–74% = 2, <65% = 3				

## Methods

A prospective observational study was conducted over a 12-month period [7]. Ethical approval was granted by the joint St James’ Hospital–Tallaght University Hospital Joint Research Ethics Committee (ID 1926). The study was registered at ClinicalTrials.gov (NCT06329479).

Patients with locally advanced or metastatic cancer were recruited from oncology and palliative care teams across a hospice and hospital setting. Inclusion criteria were: (1) Outpatient; (2) ≥18 years old; (3) Diagnosis of advanced cancer (locally advanced or metastatic, staging specific to cancer type); (4) Ambulatory; (5) Estimated prognosis ≥3 months. Exclusion criteria were: (1) Inpatient; (2) Engaged in shift work; (3) Long-haul travel in the last 14 days; (4) Cognitive impairment limiting the ability to complete the assessment tool and/or patient diary.

Participants were assessed as an outpatient over a mid-week, 5-day period using accelerometry and a study specific rest and activity diary. Wrist-accelerometry (MicroMotionlogger®, Ambulatory Monitoring Inc), was worn on the non-dominant wrist and instructions were provided on how to use the event marker to capture IN and OUT of bedtimes. The MicroMotionlogger device® demonstrates comparable assessments of sleep and wake times when compared to polysomnography [8]. The ActivPal™ device (PAL technologies) was worn on the middle and anterior aspect of the right thigh and can accurately captures sedentary activities and positional change [9, 10].

Baseline assessments (ECOG performance status [11], MSAS-SF [12], EORTC-QLQ-C30 [13], and Morningness Eveningness Questionnaire [14]) were completed at the beginning of the week, with completion of further assessments (bPSQI [6], Epworth Sleepiness Scale [15], STOP-Bang [16], and Restless Leg Criteria [17]) at the end of the week. Included questionnaires focused on symptoms, symptom-related distress, quality of life and conditions which frequently impact sleep.

Complete MSAS-SF and EORTC-QLQ-C30 questionnaires were used, which include single-item sleep questions. The MSAS-SF asks about the presence or absence of “difficulty sleeping” in the last week alongside a 5-point “distress” scale (not at all; a little bit; somewhat; quite a bit; very much). Similarly, the EORTC-QLQ-C30 asks about the presence or absence of “trouble sleeping” during the past week alongside a 4-point scale (not at all; a little; quite a bit; very much).

Wrist accelerometry data was reviewed with “bad” data, representing off-wear time, being removed. Recently published guidelines advise a monitoring period of 72-hours and thus the data was trimmed to remove the first night (first-night effect) while retaining a 72-hour period of assessment [18]. Patient-reported times of getting IN and OUT of bed were considered with preference given to Micromotionlogger® event marker timings over patient diary timings. The patient-reported timings were then adjusted to the nearest objective IN and OUT times reflected by a change from a supine to standing position (UP) or vice versa (DOWN), according to thigh accelerometry (ActivPal™).

Wrist accelerometry data, in the zero-crossing mode, was analysed using Micromotionlogger®-dedicated Action-W 2.7 and Action-4 1.16 software. This focussed on the program’s calculation of sleep efficiency (SE: 100 x sleep minutes / sleep onset-offset duration), sleep onset latency (SOL: minutes to first epoch scores as sleep), and wake after sleep onset (WASO: wake minutes during sleep onset-offset duration), with values averaged over the 72-hour period. Wrist accelerometry data was chosen for analysis to align with the most used form of accelerometry-based sleep assessments in the literature. Commonly used threshold values for abnormality, including in questionnaires, include more than 30 minutes SOL or WASO, and < 85% SE. However, normal sleep parameter values vary, particularly with age e.g. SE (35–49: 85.4%; 50–64: 83.2%; 65–79: 77.5%), WASO (35–49: 51.1 min; 50–64: 64.0 min; 65–79: 77.1 min), SOL (35–49: 14.4 min; 50–64: 15.7 min; 65–79: 19.5 min) [19]. These age-related values and generic values were considered objectively through accelerometry alongside subjective bPSQI scores to assess the impact of using different threshold values. IBM®SPSS® statistic version 29.0.1.1 (244) software was used for statistical analysis which included descriptive analysis of the data, correlational analysis, exploratory factor analysis and independent T or Mann–Whitney tests to compare objective sleep measures among sleep quality groups and linear regression modeling.

## Results

Sixty-five patients (see Table 2) completed assessments and had adequate accelerometry data for analysis. Subjective sleep disturbance was identified in 48%–60% of participants [60%–MSAS-SF; 57%–EORTC-QLQ-C30; 48%–bPSQI (score > 5)]. Objectively, 69% had long periods of wakefulness during sleep (WASO >30 minutes, 37% had a prolonged time to falling asleep (SOL >30 minutes), and 29% had proportionally less sleep between falling asleep and waking up in the morning (SE <85%).

**Table 2:** Participant baseline characteristics

Age	62 years old (SD 11.61, range 38-86)
Sex	Female (*n* = 35, 54%) Male (*n* = 30, 46%)
Cancers	Gastrointestinal (*n* = 18, 27.7%) Breast (*n* = 14, 21.5%) Lung (*n* = 13, 20%) Urological (*n* = 13, 20%) Gynecological (*n* = 4, 6.2%) Skin (*n* = 2, 3.1%) Synchronous breast/thyroid (*n* = 1, 1.5%)
Cancer extent	Metastatic (*n* = 53, 82%) Locally advanced (*n* = 8, 12%) Metastatic and locally advanced (*n* = 4, 6%) Primary tumor in-situ (*n* = 33, 51%)
European Cooperative Oncology Group (ECOG) performance status	ECOG 0 (*n* = 12, 18%) ECOG 1 (*n* = 40, 62%) ECOG 2 (*n* = 11, 17%) ECOG 3 (*n* = 2, 3%)
STOP-Bang	High risk (*n* = 8, 12%) Intermediate risk (*n* = 25, 39%) Low risk (*n* = 32, 49%)
Restless Leg Syndrome criteria	Met (*n* = 11, 17%) Not met (*n* = 54, 83%)
Chronotype (Morningness Eveningness Questionnaire, MEQ)	Definite morning (*n* = 6, 9%) Moderate morning (*n* = 24, 37%) Intermediate (*n* = 33, 51%) Moderate evening (*n* = 2, 3%)
Epworth Sleepiness Scale score	Median score 4 (range 0–21) Lower normal levels (*n* = 39, 60%) Higher normal levels (*n* = 18, 28%) Mild excessive levels (*n* = 5, 8%) Moderate excessive levels (*n* = 1, 1%) Severe levels (*n* = 2, 3%)
Mood (MSAS-SF)	Feeling sad (*n* = 31, 48%) Worrying (*n* = 31, 48%) Feeling irritable (*n* = 29, 45%) Feeling nervous (*n* = 16, 25%)
Sleeping arrangements	Own bedroom (*n* = 34, 52%) Shared bedroom (*n* = 31, 48%)

The bPSQI global scores were considered against subjective and objective assessments of sleep (Table 3). The mean bPSQI global score differed for those with and without single-item subjective sleep complaints (MSAS-SF: 7.44 +/– 3.91 vs. 3.23 +/– 2.07, *p*<0.001; EORTC-QLQ-C30: 7.59 +/– 3.78 vs. 3.32 +/– 2.42, *p*<0.001). However, bPSQI scores did not differ between those with and without objective accelerometry-derived sleep disturbance based on generic, or age-standardized, threshold values for SE, SOL or WASO (all *p*>0.05).

**Table 3:** Comparison of bPSQI global scores between subjective single-item questions, sleep-related distress, and objective accelerometry assessments of sleep

Assessment	bPSQI >5	bPSQI ≥8
**Questionnaire**		
***MSAS-SF (Yes n = 39)***	*n* = 30(77%)	*n* = 23 (60%)
Score 0-1 (Distress: not at all, a little) (*n* = 14)	*n* = 8 (57%)	*n* = 6 (43%)
Score 2-4 (Distress: Somewhat–Very much) (*n* = 25)	*n* = 23 (92%)	*n* = 17 (68%)
***EORTC-QLQ-C30 (Yes, n = 37)***	*n* = 29 (78%)	*n* = 21 (57%)
Score 2 (A little) (*n* = 12)	*n* = 7 (58%)	*n* = 3 (25%)
Score 3-4 (Quite a bit–Very much) (*n* = 25)	*n* = 22 (88%)	*n* = 18 (72%)
***Accelerometry***		
Normal sleep efficiency, ≥85% (*n* = 46)	*n* = 25 (54%)	*n* = 15 (33%)
Abnormal sleep efficiency, <85% (*n* = 19)	*n* = 10 (53%)	*n* = 8 (42%)
Normal age-adjusted sleep efficiency (*n* = 49)	*n* = 27 (55%)	*n* = 17 (35%)
Abnormal age-adjusted sleep efficiency (*n* = 16)	*n* = 8 (50%)	*n* = 6 (38%)
Normal sleep onset latency, ≤30 min (*n* = 41)	*n* = 25 (70%)	*n* = 19 (46%)
Abnormal sleep onset latency, >30 min (*n* = 24)	*n* = 10 (42%)	*n* = 4 (17%)
Normal age-adjusted sleep onset latency (*n* = 27)	*n* = 13 (48%)	*n* = 11 (41%)
Abnormal age-adjusted sleep onset latency (*n* = 38)	*n* = 22 (58%)	*n* = 12 (32%)
Normal wake after sleep onset, ≤30 min (*n* = 20)	*n* = 8 (40%)	*n* = 6 (30%)
Abnormal wake after sleep onset, >30 min (*n* = 45)	*n* = 28 (62%)	*n* = 18 (40%)
Normal age-adjusted wake after sleep onset (*n* = 45)	*n* = 24 (53%)	*n* = 14 (31%)
Abnormal age-adjusted wake after sleep onset (*n* = 20)	*n* = 11 (55%)	*n* = 9 (45%)

Simple linear regression modeling demonstrated that SE, WASO and SOL played a small and non-significant role in predicting bPSQI scores. Sleep disturbance identified by MSAS-SF and EORTC-QLQ-C30 was predictive of bPSQI scores [bPSQI score = 3.23 + 4.21 (if MSAS-SF “difficulty sleeping” present) and (bPSQI score = 3.32 + 4.27 (if EORTC-QLQ-C30 “trouble sleeping” present))]. This may suggest a baseline score of 3 or less is found in those without sleep complaints and a score of 8 or more found in those with sleep complaints. Scores of 4-7 may be indeterminate. When using these values, those with poor sleep quality (bPSQI ≥8) versus those without poor sleep quality (bPSQI ≤3) had non-significantly longer sleep onset latency and wake after sleep onset, and lower sleep efficiency (all *p*>0.05).

Patients were more likely to score positively at either threshold value (bPSQI >5 or ≥ 8) with increasing self-reported sleep-related distress. Patients were also more likely to score positively on the bPSQI if SOL (objective) was adjusted for age and less likely to score positively when SE (objective) was adjusted for age.

The bPSQI items demonstrated no strong floor or ceiling effect and strong correlations were seen between items (*p*<0.001). Good internal consistency was demonstrated (Cronbach’s alpha 0.751). Internal consistency was only improved by removal of the item addressing waking in the middle of the night or early morning (Cronbach’s alpha 0.785). Following exploratory factor analysis, only factor 1 (sleep quality) had an Eigenvalue >1 and explained 55.36% of the variance.

Significant correlations were noted between subjective measures and between objective measures (see Table 4). No significant correlations were noted between subjective and objective measures.

**Table 4:** Correlations between subjective and objective measures of sleep

		1	2	3						4		
				a	b	c	d	e	f	a	b	c
1. MSAS “difficulty sleeping”		–	–	–	–	–	–	–	–	–		
2. EORTC “trouble sleeping”		**0.875 (*p*<0.001)**	–	–	–	–	–	–	–			
bPSQI	a. GS	**0.524 (*p*<0.001)**	**0.547 (*p*<0.001)**	–	–	–	–	–	–			
	b. SOL	**0.461 (*p*<0.001)**	**0.465 (*p*<0.001)**	**0.592 (*p*<0.001)**	–	–	–	–	–			
	c. SDur	**0.436 (*p*<0.001)**	**0.428 (*p*<0.001)**	**0.703 (*p*<0.001)**	**0.359 (*p*=0.003)**	–	–	–	–			
	d. SE	**0.372 (*p*=0.02)**	**0.419 (*p*<0.001)**	**0.769 (*p*<0.001)**	**0.333 (*p*=0.007)**	**0.599 (*p*<0.001)**	–	–	–			
	e. SDist	**0.318 (*p*=0.010)**	**0.316 (*p*=0.010)**	**0.711 (*p*<0.001)**	0.220 (*p*=0.079)	0.222 (*p*=0.076)	**0.320 (*p*=0.009)**	–	–			
	f. SQ	**0.392 (*p*=0.001)**	**0.399 (*p*<0.001)**	**0.782 (*p*<0.001)**	**0.467 (*p*<0.001)**	**0.537 (*p*<0.001)**	**0.506 (*p*<0.001)**	**0.507 (*p*<0.001)**	–			
4. Objective accelerometry	a. SOL	−0.226 (*p*=0.070)	−0.204 (*p*=0.104)	0.004 (*p*=0.976)	−0.066 (*p*=0.600)	−0.109 (*p*=0.387)	0.078 (*p*=0.538)	0.059 (*p*=0.641)	−0.066 (*p*=0.603)	–	–	–
	b. SE	0.053 (*p*=0.677)	0.094 (*p*=0.459)	−0.028 (*p*=0.825)	0.091 (*p*=0.469)	0.169 (*p*=0.178)	−0.169 (*p*=0.179)	−0.77 (*p*=0.540)	0.047 (*p*=0.709)	**−0.287 (*p*=0.020)**	–	—
	c. WASO	−0.031 (*p*=0.807)	−0.080 (*p*=0.525)	0.055 (*p*=0.661)	−0.061 (*p*=0.628)	−0.205 (*p*=0.102)	0.219 (*p*=0.079)	0.100 (*p*=0.428)	−0.21 (*p*=0.868)	**0.246 (*p*=0.048)**	**−0.978 (*p*<0.001)**	–

Abbreviations: GS, global score; SOL, sleep onset latency; SD, sleep duration; SE, sleep efficiency; S WASO, wake after sleep onset.

The MSAS-SF single question (“difficulty sleeping”) and EORTC-QLQ-C30 (“trouble sleeping”) demonstrated good accuracy at identifying poor sleep quality (bPSQI >5 vs. ≤5) (MSAS-SF: sensitivity 90.3%; EORTC-QLQ-C30: sensitivity 84.4%), but low accuracy at identifying those without bPSQI-identified poor sleep (MSAS-SF: specificity 67.6%; EORTC-QLQ-C30: specificity 66.7%). Similar results were found with the adjusted threshold values for identifying poor sleep quality (bPSQI ≥8 vs. ≤3) (MSAS-SF: sensitivity 95.7%, specificity 59.1%; EORTC-QLQ-C30: sensitivity 87.0%, specificity 60.9%).

## Discussion

This is the first study to consider the use of the bPSQI in patients with advanced cancer. The bPSQI demonstrates good internal consistency, agreement with single-item sleep questions and increasing scores with increasing patient reported distress. bPSQI global scores largely relate to self-reported sleep quality. Prevalence of sleep disturbance varied depending on the measure used. Although a relationship was noted between subjective measures of sleep disturbance, the bPSQI scores were not significantly associated with objective accelerometry evidence of sleep disruption. This finding is supported by previous studies of patients with different cancer diagnoses (including those with advanced cancer) in the inpatient and outpatient setting, along with their careers, that have demonstrated discrepancies in detecting poor sleep using several objective sleep parameters (SOL, TST, SE, WASO) and the PSQI [20–22]. Furthermore, this study highlights a bPSQI cut-off score of 8, rather than 5, may be more accurate at detecting subjective sleep disturbance in patients with advanced cancer. This finding is similar to previous work which evaluated the psychometric properties of the full PSQI and noted a cut-off of >8, rather than >5, being more accurate in cancer patients [5]. Single-item questions have previously demonstrated strong associations with scores from the PSQI, Medical Outcomes Study Sleep Scale, and the Jenkins Sleep Scale [23, 24]. Although the study highlights that single-item questions within the MSAS-SF and EORTC-QLQ-C30 can accurately identify those with bPSQI-identified poor sleep, they are at risk of incorrectly identifying poor sleep when not present. The findings in this study are limited by the small sample size but support further research to assess the validity of the bPSQI, including assessment of convergent validity against alternate sleep-specific validated tools. If proven valid, the bPSQI offers a quick and acceptable tool to assess for sleep disturbance. Due to discrepancies in subjective and objective measures of sleep, future studies should ensure that the outcome measures are appropriately matched with the aims of the study.

## Data Availability

Data are available on reasonable request. Requests for data sharing should be made to the Academic Department of Palliative Medicine in Dublin (adpm@olh.ie).

## Declarations

Ethics approval and consent to participate: Ethical approval was granted by the joint St James’ Hospital–Tallaght University Hospital Joint Research Ethics Committee (ID 1926). The study was registered at ClinicalTrials.gov (NCT06329479). Participants were required to provide informed consent.
